# Microplanning workshops for high-quality vaccination in Brazil: an experience report, 2023

**DOI:** 10.1590/S2237-96222024v33e2024046.en

**Published:** 2024-12-16

**Authors:** Ana Catarina de Melo Araújo, Luciana Maiara Diogo Nascimento, Carla Conceição Ferraz, Elice Eliane Nobre Ribeiro, Fernanda Penido Matozinhos, Eder Gatti Fernandes

**Affiliations:** 1 Ministério da Saúde, Secretaria de Vigilância em Saúde e Ambiente, Brasília, DF, Brazil; 2 Observatório de Pesquisa e Estudos em Vacinação, Belo Horizonte, MG, Brazil; 3 Universidade Federal de Minas Gerais, Escola de Enfermagem, Belo Horizonte, MG, Brazil; 4 Ministério da Saúde, Secretaria de Vigilância em Saúde e Ambiente, Brasília, DF, Brazil

**Keywords:** Vacunación, Cobertura de Vacunación, Programa de Inmunización, Microplanificación, Planificación de la Salud, Vaccination, Vaccination Coverage, Immunization Program, Microplanning, Health Planning

## Abstract

**Objective:**

To report the experience of workshops designed for action plans in microplanning vaccination in Brazilian states and municipalities.

**Methods:**

This was a report on microplanning workshops aimed at mapping the local population, target population and identifying appropriate and effective vaccination actions. Vaccination actions were planned according to the reality of the municipalities using the microplanning method for high-quality vaccination activities.

**Results:**

The workshops aimed at establishing effective vaccination strategies were held between July 15 (Macapá, capital city of Amapá state) and September 14, 2023 (Belo Horizonte, capital city of the state of Minas Gerais), with a total of 1,232 participants, including surveillance technicians, primary care and other health sectors. The diversity of participants highlighted distinct challenges and the importance of cooperation in addressing low vaccination coverage across the country.

**Conclusion:**

The workshops served as a platform for raising awareness and exchanging experiences among stakeholders involved in vaccination.

## Study contributions

**Main results:**

The workshops promoted detailed mapping of the local context and the target population, in addition to providing opportunities for raising awareness and exchanging experiences among participants involved in vaccination programs.

**Implications for services:**

It is suggested that the workshops be replicated and expanded to promote a continuous and contextualized assessment of local realities. The process also fosters a more collaborative and participatory approach, enabling professionals to adjust their practices based on local demands and actual needs.

**Perspectives:**

The next steps are expected to include the sustainable incorporation of this strategy across states and municipalities, in order to eliminate and control vaccine-preventable diseases.

## RESUMEN

**Objetivo:**

Informar sobre la experiencia de talleres de microplanificación de planes de acción para la vacunación en estados y municipios brasileños.

**Métodos:**

Este es un informe sobre talleres de microplanificación para mapear la realidad local, la población objetivo e identificar acciones de vacunación apropiadas y eficaces. Las acciones de vacunación se planificaron de acuerdo con la realidad de los municipios utilizando el método de microplanificación de actividades de vacunación de alta calidad.

**Resultados:**

Los talleres destinados a establecer estrategias eficaces de vacunación se realizaron entre el 15 de julio (Macapá) y el 14 de septiembre de 2023 (Belo Horizonte), con un total de 1.232 participantes, entre técnicos de vigilancia, atención primaria y otros sectores de la salud. La diversidad de los participantes reveló diferentes desafíos y la importancia de la cooperación para hacer frente a la baja cobertura de vacunación en el país.

**Conclusión:**

Los talleres demostraron ser un espacio de sensibilización e intercambio de experiencias entre los actores implicados en la vacunación.

## INTRODUCTION

Vaccination in Brazil is coordinated by the National Immunization Program (*Programa Nacional de Imunizações* - PNI) under the Ministry of Health. This program is globally recognized for its complexity and has historically ensured high vaccination coverage for the Brazilian population.^1^ In recent years, a decrease in vaccination coverage for several immunobiological agents,^6 -7^ has been observed in Brazil and other countries.^2 -5^

The microplanning strategy for high-quality vaccination activities has been implemented over the past two decades by local immunization professionals in countries across the Americas with positive outcomes.^8^ Microplanning is used as a strategy to ensure high-quality vaccination activities, such as routine immunization, campaigns, intensification, sweeps and house-to-house vaccination, by enabling changes in the work process.

Understanding the planning of multi-vaccination actions should take into account high-quality vaccination activities and microplanning, according to the adapted Plan for the Implementation of High-Quality Vaccination Activities and the Microplanning Process – Routine and Intramural and Extramural Vaccination Program, from the Pan American Health Organization (PAHO). The Minister of Health’s Ordinance No. 844, of July 14, 2023,^9^ established guidelines for multi-vaccination actions within the Brazilian National Health System during 2023. This included the establishment of exceptional and temporary financial incentives to support multi-vaccination actions in municipalities, states and the Federal District, aiming to increase vaccination coverage among children and adolescents up to 15 years of age in the country. The ordinance also guides municipalities to incorporate microplanning strategies for high-quality vaccination activities in their municipal health plans.

Workshop designed for action plans in microplanning were then developed and conducted as a strategy for mapping the local reality and target population and identifying appropriate and effective vaccination actions. Vaccination actions were planned according to the reality of the municipalities using the microplanning method for high-quality vaccination activities.

Given the relevance and utility of these tools, the objective of this study was to report on the experience of action plan workshops by Brazilian states and municipalities.

## METHODS

This was an experience report on workshops held in 2023. Given the need to reverse the decline in vaccination coverage as of January 1, 2023, changes were implemented within the Ministry of Health regulatory structure, including the reformulation and expansion of the PNI, which was elevated from a general coordination to a department, with four new general coordinations.

Microplanning for high-quality vaccination activities was chosen as the strategy to increase vaccination coverage, introduced by PAHO as an approach for promoting differentiated vaccination actions.

The following stages were considered in the development of the workshops, aligned with the Minister of Health’s Ordinance No. 844, dated July 14, 2023:^9^

- Stage 1: analysis of the health situation, which included organizing data, mapping and segmenting localities, in order to identify the susceptible population and the availability of vaccination services.
- Stage 2: planning and programming, which involved defining vaccination strategies and communication and social mobilization plans and calculating the needs for the action, based on the previous stage’s mapping – this included vaccines, syringes, human resources, general materials, the cold chain, and performance analysis.
- Stage 3: follow-up and supervision with rapid vaccination monitoring, in order to identify pockets of susceptible individuals, people pending vaccination and the implementation of interventions.
- Stage 4: supervision and evaluation to monitor progress in achieving the vaccination targets.

The implementation was carried out according to the methodology outlined below, with an approximate total duration of 24 hours, spread over three days. Initially, the participants of each workshop were divided into groups, organized by affinity, according to health regions, levels of operation, healthcare facilities, and other aspects. The role of facilitators during the process was fulfilled by the technical team from the Ministry of Health.

The first session involved presenting the epidemiological situation and vaccination coverage in Brazil. Subsequently, topics covered included: guidelines for microplanning and vaccination actions; high-quality components and criteria; the preparation and formation of the planning and execution committee; training, qualification and updates for vaccination actions; microplanning and execution stages; analysis of the health situation; considerations for reaching vulnerable populations; vaccination records and perspectives of the Ministry of Health’s information systems; vaccination concepts and indicators; method for calculating the unvaccinated cohort; assessment of the vaccination activity preparedness; safe vaccination planning and organizing the monitoring of events supposedly attributable to vaccination or immunization; communication and social mobilization for vaccination actions; monitoring and supervision of microplanning; and rapid vaccination assessment and monitoring and evaluation indicators.

The workshops were led by a coordinator and approximately 10 facilitators. The central coordinating group followed the team discussions, providing clarifications and sharing experiences and proposing joint actions. During the workshop, documents relevant to organizing microplanning in municipalities were presented in an interactive format. This sharing of work processes played a crucial role in identifying health needs and, consequently, represented an opportunity for improving the responsiveness of service.^10- 12^

Two indicators were developed to assess the distribution of scores based on the evaluation of the workshops held in each state, using a tool sent by the Ministry of Health after the Workshops: (i) an average score from 1 to 5, obtained by a simple mean (answered by the participants and divided by the number of responses received). A higher score indicated a better evaluation. Scores 4 and 5 were considered good and excellent, respectively; and (ii) participant satisfaction percentage regarding the total number of respondents.

This study was approved by the Research Ethics Committee of the Universidade Federal de Minas Gerais under opinion No. 6,739,296, dated 2/4/2024, certificate of submission for ethical appraisal 78076124.8.0000.5149.

## RESULTS

A total of 13 workshops were conducted between July and September 2023, with 1,232 participants from immunization teams, technicians from indigenous health districts, primary care and surveillance technicians from capital cities, all of whom would implement microplanning in their territories. In some workshops, technicians from municipalities that serve as regional hubs, such as São Paulo, also participated. Four states had technicians from all (or nearly all) of their municipalities participating in the workshops offered by the Ministry of Health: Amazonas, Acre, Amapá, and Roraima.

The pilot workshop took place in Natal, the capital city of Rio Grande do Norte state, with representatives from the state and 16 municipalities, in response to the need to intensify vaccination against yellow fever due to epizootics registered in the region, funded and organized by PAHO. Following the experience in Rio Grande do Norte, the Coordination Committee for High-Quality Vaccination Activities was established, comprising representatives from the PNI, the Secretariat of Primary Health Care Secretariat (PHC) and the Special Indigenous Health Secretariat, and adjustments were made to the workshop and support materials provided by PAHO. As a result, microplanning workshops and the advancement of multi-vaccination were offered to two states (Amazonas and Acre) and their municipalities. Prior to the publication of this ordinance, two workshops were held in Amazonas state and two workshops in Acre state, with the participation of technicians from the states and all municipalities.

**Figure 1:**
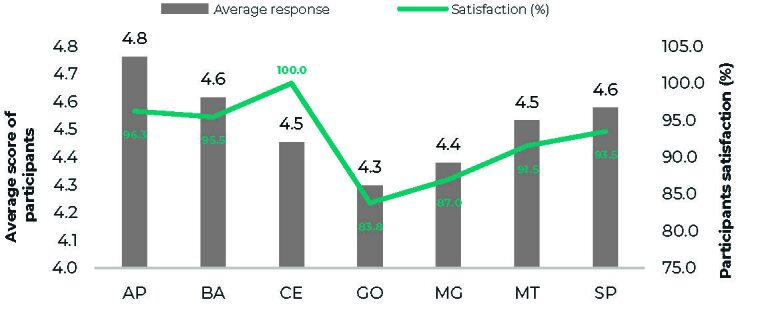
Evaluation score of the workshops conducted in each state, Brazil, 2023

The workshop with the largest number of participants was in Manaus state (13.3%), followed by São Paulo (12.7%) and Curitiba (8.7%) states (Table 1). The workshop in Goiás state, which included participants from Goiás, Tocantins and Rondônia, had the lowest average score (4.30) among all the states (Figure 1). Despite this, 83.8% rated the 22 items as good or excellent. The workshop with the highest average score was in Amapá (conducted with the state of Amapá and its municipalities), with a score of 4.76 and a satisfaction rate of 96.3%. The workshop in Ceará state (with participants from Alagoas, Piauí, Ceará and Paraíba states) had an average score of 4.45, but 100% satisfaction rate. This indicates that all participants rated it 4 or 5, that is, good and excellent, but more chose 4 than 5.

**Table 1:** Distribution of participants in the workshops by Federative Unit (FU), Brazil, 2023

Workshop number	Dates	Location	Participants’ FU	Participants by FU (n)	Total participants, n (%)
1	April 24-28	Natal	Rio Grande do Norte	68	68 (5.5)
2	May 8^th^ to 12^th^	Manaus	Amazonas	158	164 (13.3)
			Roraima and São Paulo	6	
3	May 18-26	Cruzeiro do Sul and Rio Branco	Acre	89	93 (7.5)
			Rondônia	4	
4	July 10-13	Macapá	Amapá	63	67 (5.4)
			Maranhão	1	
			Rondônia	3	
5	July 18-20	Belém	Roraima	18	67(5.4)
			Maranhão	24	
			Pará	25	
6	July 25-27	Vitória	Rio de Janeiro	31	68 (5.5)
			Espírito Santo	20	
			Distrito Federal	15	
			Rio Grande do Sul	2	
7	July 31^st^ to August 4^th^	São Paulo	São Paulo	156	156(12.7)
8	August 8^th^ to 10^th^	Cuiaba	Mato Grosso	44	67 (5.4)
			Mato Grosso do Sul	21	
			Minas Gerais	2	
9	August 15-17	Curitiba	Paraná	41	107 (8.7)
			Santa Catarina	25	
			Rio Grande do Sul	36	
			Rio Grande do Norte	3	
			Minas Gerais	2	
10	August 22-24	Fortaleza	Alagoas	15	77 (6.3)
			Piauí	20	
			Ceará	20	
			Paraíba	20	
			Rio Grande do Norte	2	
11	August 29-31	Goiania	Distrito Federal	1	101 (8.2)
			Goiás	57	
			Tocantins	28	
			Rondônia	15	
12	September 4^th^ to 6^th^	Salvador	Acre	1	96 (7.8)
			Bahia	54	
			Pernambuco	25	
			Rio Grande do Norte	2	
			Sergipe	14	
13	September 14^th^ to 14^th^	Belo Horizonte	Amazonas	2	101 (8.2)
			Minas Gerais	98	
			Rio Grande do Sul	1	

**Figure 2:**
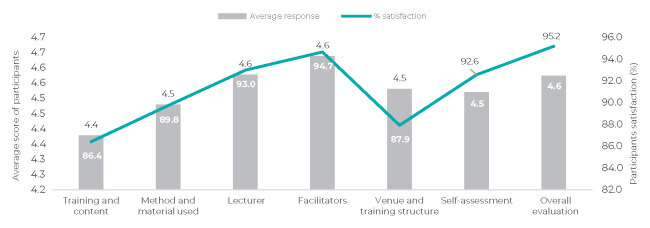
Objective assessment of the states where the workshops were conducted, by dimension, Brazil, 2023

The lowest average score among the dimensions was for training and content, with a score of 4.38 (Figure 2). However, the satisfaction rate was high, with 86.4% of participants rating it as good or excellent. There was a slight difference compared to the dimension that had the highest score: facilitators, with a score of 4.64. Satisfaction was 94.7%. The overall evaluation of the training was 4.57, an item that does not take the other items into consideration, with 95.2% satisfaction, marking it as good or excellent.

## DISCUSSION

Between July and September 2023, 13 microplanning workshops were conducted with 1,232 participants from immunization teams, indigenous health technicians, primary care, and surveillance from various regions. States such as Amazonas, Acre, Amapá, and Roraima had broad participation from their municipalities, and the workshop held in Manaus recorded the highest number of participants (13.3%). The pilot workshop took place in Rio Grande do Norte state, focusing on intensifying yellow fever vaccination. Overall evaluations were positive, with an average score of 4.57 and 95.2% satisfaction, with facilitators receiving the highest score (4.64) and training and content receiving the lowest score (4.38).

In Brazil, the processes of demographic and epidemiological transitions are interconnected phenomena, occurring alongside the country’s social and economic development, with varying effects on the population’s health across macro-regions. ^12^ Workshops were conducted in different states of Brazil and offered to all 27 Federative Units. The highest number of sessions occurred in August 2023, totaling six, with 578 participants. During this month, the trainings were held in São Paulo, Mato Grosso do Sul, Paraná, Ceará and Goiás - some held the workshop jointly, due to geographic proximity.

The expansion of PHC in Brazil is significant, with an increase in the number of Primary Health Care Centers, greater population coverage by the Family Health Strategy and increased access, ^14- 15^ including in immunization. However, regional disparities and political challenges persist in Brazil, affecting and contributing to the decline in vaccination coverage.

Microplanning has led to effective changes in the systematization of activities, such as the routine program, intensification and extramural vaccination (which had already been practiced by immunization professionals at the local level, although undocumented). This method enabled the incorporation of criteria and indicators into services.^8^

The stages of microplanning are dynamic, allowing for dialectical processes to discuss the challenges and easiness in immunization efforts that can contribute to improving vaccination coverage. States and municipalities are expected to develop microplanning action plans based on their local realities and the target population to identify the most appropriate and effective intra-and extramural vaccination activities.

High-quality vaccination activities corroborate and meet the criteria developed by PAHO using the methodology of productive health service management. It is worth highlighting that the aforementioned criteria encompass the key pillars of efficacy, homogeneity, timeliness and efficiency. This approach plays a central role as a strategy to meet targets, ensure accurate need assessment, optimize available resources, and guarantee widespread population access to vaccination. ^16^

The workshops generated discussions and it is expected that state and municipal managers will understand the importance of strengthening, incorporating and prioritizing vaccination actions within the routine of health services. In the long term, the sustainable incorporation of the strategy by Brazilian states and municipalities is expected to eliminate and control vaccine-preventable diseases. The impact of its implementation has been observed in increased vaccination coverage and the increase in the number of municipalities meeting vaccination targets, comparing 2022 (pre-microplanning) and 2023 (post-microplanning), especially for the adsorbed diphtheria, tetanus and pertussis vaccine, hepatitis A and the MMR vaccine. This reflects improvement compared to previous practices, which showed concerning drops in vaccination coverage.^16^ The workshops were important in facilitating experience-sharing among the various players involved, fostering integration across different management levels, aligned with local needs.^16^

The diversity of vaccination professionals was able to highlight key challenges faced by states and municipalities in achieving and maintaining adequate vaccination coverage. It is essential that internal and external stakeholders are engaged in the microplanning process in order to enable moments of reflection and cooperation capable of generating knowledge and change health practices, in addition to increasing understanding of critical processes that may be related to low vaccination coverage in Brazil. ^17^

The need for interaction and shared responsibility between PHC and epidemiological surveillance teams, as well as support between states and municipalities are highlighted. This was one of the main points observed from the analysis of this study, having been thoroughly discussed during the workshops. This alignment is crucial for the control of vaccine-preventable diseases. ^18^

As a limitation, the short period between the implementation of the workshops and the evaluation of their impacts stands out. It is worth mentioning that these findings may not reflect long-term changes in microplanning actions. Another limitation was the reliance on participants’ self-assessment, which, although useful, may introduce a positive response bias.

The microplanning workshops proved to be a space for raising awareness and exchanging experiences among those involved in vaccination. These issues were able to spark discussions and are expected to lead to the incorporation of actions into the routine of health services. This provides evidence to support high-quality vaccination activities and a permanent vaccination regimen in these contexts, which includes the logistical and operational aspects necessary for the successful implementation of such strategy. Completing and discussing data collection tools during the workshops made it possible to bridge the gap between theory and practice, in addition to promoting qualified input of participants and reporting on the needs faced in health services.
