# Supplementary material for: Microplanning workshops for high-quality vaccination in Brazil: an experience report, 2023
Source: Epidemiol Serv Saude. 2024 Dec 16;33:e2024046. doi: 10.1590/S2237-96222024v33e2024046.en (PMC11654813; doi:10.1590/S2237-96222024v33e2024046.en)
Supplement: Translation

# Translation: Oficinas de microplanejamento para vacinação de alta qualidade no Brasil: relato de experiência, 2023

**DOI:** 10.1590/S2237-96222024v33e2024046.pt

**Authors:** Ana Catarina de Melo Araújo, Luciana Maiara Diogo Nascimento, Carla Conceição Ferraz, Elice Eliane Nobre Ribeiro, Fernanda Penido Matozinhos, Eder Gatti Fernandes

**Affiliations:** 1 Ministério da Saúde, Secretaria de Vigilância em Saúde e Ambiente, Brasília, DF, Brasil; 2 Observatório de Pesquisa e Estudos em Vacinação, Belo Horizonte, MG, Brasil; 3 Universidade Federal de Minas Gerais, Escola de Enfermagem, Belo Horizonte, MG, Brasil; 4 Ministério da Saúde, Secretaria de Vigilância em Saúde e Ambiente, Brasília, DF, Brasil

**Keywords:** Vacinação, Cobertura Vacinal, Programa de Imunizações, Microplanejamento, Planejamento em Saúde

## Abstract

**Objetivo:**

Relatar a experiência das oficinas do plano de ações de microplanejamento para vacinação em estados e municípios brasileiros.

**Métodos:**

Trata-se de um relato sobre oficinas de microplanejamento para mapeamento da realidade local, população-alvo e identificação das ações de vacinação adequadas e eficazes. Planejaram-se ações de vacinação de acordo com a realidade dos municípios por meio do método de microplanejamento para as atividades de vacinação de alta qualidade.

**Resultados:**

As oficinas com objetivo de estabelecer estratégias eficazes de vacinação foram realizadas entre 15 de julho (Macapá) a 14 de setembro de 2023 (Belo Horizonte), totalizando 1.232 participantes, dentre eles, técnicos de vigilância, atenção primária e outros setores da saúde. A diversidade dos participantes revelou desafios distintos e a importância da cooperação para enfrentar as baixas coberturas vacinais no país.

**Conclusão:**

As oficinas mostraram-se um espaço na sensibilização e na troca de experiências entre atores envolvidos com a vacinação.

## Contribuições do estudo

**Principais resultados:**

As oficinas promoveram o mapeamento detalhado da realidade local e da população-alvo, além de proporcionar oportunidades de sensibilização e troca de experiências entre os participantes envolvidos nos programas de vacinação.

**Implicações para os serviços:**

Sugere-se replicar e expandir as oficinas para promover uma avaliação contínua e contextualizada das realidades locais. O processo também fomenta uma abordagem mais colaborativa e participativa, permitindo que os profissionais ajustem suas práticas com base nas demandas locais e nas necessidades reais.

**Perspectivas:**

Espera-se, como próxima ação, a incorporação da estratégia de forma sustentável nos estados e municípios, a fim de eliminar e controlar doenças preveníveis por vacinação.

## INTRODUÇÃO

A vacinação no Brasil é coordenada pelo Programa Nacional de Imunizações (PNI), do Ministério da Saúde. Tal programa é reconhecido mundialmente por destacar pelo seu grau de complexidade e, historicamente, garantir altas coberturas vacinais para a população brasileira.^1^ Nos últimos anos, no Brasil e em outros países,^2 -5^ verificou-se queda na cobertura vacinal de vários imunobiológicos.^6 -7^

A estratégia de microplanejamento para as atividades de vacinação de alta qualidade é um processo que vem sendo realizado nas últimas duas décadas por profissionais da imunização do nível local dos países da região das Américas com desfechos positivos.^8^ Para operacionalizar as atividades de vacinação de alta qualidade, utiliza-se do microplanejamento como estratégia para garantir mudanças nos processos de trabalho e a alta qualidade de atividades, como vacinação de rotina, campanhas, intensificações, varreduras e vacinação casa a casa. 

A compreensão do planejamento das ações da multivacinação deverá considerar as atividades de vacinação de alta qualidade e o microplanejamento, conforme a adaptação do Plano para Implementação das Atividades de Vacinação de Alta Qualidade e do Processo de Microplanejamento – Programa de Rotina e Vacinação Intramuros e Extramuros, da Organização Pan-Americana da Saúde (OPAS). Por meio da Portaria Gabinete do Ministro do Ministério da Saúde nº 844, de 14 de julho de 2023,^9^ estabeleceu diretrizes para a realização das ações de multivacinação no contexto do Sistema Único de Saúde durante 2023. Isso abrangeu a instituição de incentivo financeiro de custeio, excepcional e temporário, para ações de multivacinação nos municípios, nos estados e no Distrito Federal, visando ampliar a cobertura vacinal entre crianças e adolescentes de até 15 anos de idade no país. A portaria também orienta os municípios a incorporarem estratégia de microplanejamento para atividades de vacinação de alta qualidade nos seus planos municipais de saúde. 

Foram, então, planejadas e realizadas oficinas do plano de ações de microplanejamento como estratégia para mapeamento da realidade local e população-alvo e identificação das ações de vacinação adequadas e eficazes. Planejaram-se ações de vacinação de acordo com a realidade dos municípios por meio do método de microplanejamento para as atividades de vacinação de alta qualidade.

Diante da relevância e utilidade dessas ferramentas, objetivou-se relatar a experiência das oficinas do plano de ações por estados e municípios brasileiros.

## MÉTODOS

Trata-se de relato de experiência de oficinas realizadas em 2023. Diante da necessidade de reverter a queda das coberturas vacinais a partir 1º de janeiro de 2023, implantaram-se mudanças na estrutura regimental do Ministério da Saúde, incluindo a reformulação e a ampliação do PNI, que passou de coordenação-geral para departamento, com quatro novas coordenações-gerais.

Optou-se pelo microplanejamento para as atividades de vacinação de alta qualidade com o objetivo de aumentar as coberturas vacinais, trazido pela OPAS como estratégia para promoção de ações de vacinação diferenciadas. 

As seguintes etapas foram consideradas na construção das oficinas, em alinhamento com a Portaria Gabinete do Ministro do Ministério da Saúde nº 844, de 14 de julho de 2023:^9^

- Etapa 1: análise da situação de saúde, o que inclui a organização dos dados e o mapeamento e setorização das localidades, a fim de identificar a população suscetível e a disponibilidade dos serviços de vacinação.
- Etapa 2: planejamento e programação, com a definição das estratégias para a vacinação e o plano de comunicação e mobilização social; cálculo das necessidades para a ação, a partir do mapeamento já feito na etapa anterior: vacinas, seringas, recursos humanos, materiais em geral e cadeia de frio e a análise do desempenho.
- Etapa 3: seguimento e supervisão com o monitoramento rápido de vacinação, que deverá ser realizado para identificar os bolsões de suscetíveis, as pessoas pendentes para vacinação e a execução de intervenções.
- Etapa 4: supervisão e avaliação para o monitoramento dos avanços relacionados ao cumprimento das metas.

A operacionalização ocorreu de acordo com a metodologia abordada a seguir, com carga horária aproximada de 24 horas, divididas em três dias. Primeiramente, os participantes de cada oficina foram divididos em grupos, organizados por afinidade, segundo as regiões de saúde, nível de atuação, estabelecimento de saúde, dentre outros aspectos. O papel de facilitadores durante o processo coube à equipe técnica do Ministério da Saúde. 

No primeiro momento, foi realizada a apresentação sobre a situação epidemiológica e das coberturas vacinais no Brasil. Posteriormente, abordaram-se temas como: diretrizes para o microplanejamento e ações de vacinação; componentes e critérios de alta qualidade; preparação e formação de comitê de planejamento e execução; formação, capacitação e atualização para as ações de vacinação; microplanejamento e etapas de execução; análise da situação de saúde; considerações para alcançar populações vulneráveis; registro de vacina e perspectivas dos sistemas de informação do Ministério da Saúde; conceitos e indicadores de vacinação; método de cálculo da coorte de não vacinados; avaliação da preparação das atividades de vacinação; planejamento da vacinação segura e organização do monitoramento de eventos supostamente atribuíveis à vacinação ou imunização; comunicação e mobilização social para as ações de vacinação; seguimento e supervisão do microplanejamento; e avaliação e monitoramento rápido de vacinação e indicadores de avaliação.

As oficinas foram conduzidas por um coordenador e em torno de 10 facilitadores. O grupo condutor central acompanhou as discussões das equipes, esclarecendo dúvidas e socializando a troca de experiências e a proposição de ações conjuntas. Durante os dias de oficina, foram apresentados, em exposição dialogada, os documentos a serem considerados durante a organização do microplanejamento pelos municípios. Reforça-se a importância desse momento de compartilhamento dos processos de trabalho na identificação das necessidades de saúde e, consequentemente, como oportunidade de melhoria de resposta dos serviços.^10- 12^

Dois indicadores foram construídos para avaliar, por meio de instrumento enviado pelo Ministério da Saúde após as oficinas, a distribuição das notas da avaliação das oficinas realizadas em cada estado: (i) a nota média de 1 a 5, obtida pela média simples (respondida pelos participantes e dividido pelo número de respostas recebidas). Quanto maior a nota, melhor a avaliação. Notas 4 e 5 foram consideradas como bom e excelente, respectivamente; e (ii) percentual da satisfação dos participantes, em relação ao total de respondentes. 

Este estudo foi aprovado pelo Comitê de Ética e Pesquisa da Universidade Federal de Minas Gerais por meio do parecer 6.739.296, de 2/4/2024, certificado de apresentação de apreciação ética 78076124.8.0000.5149.

## RESULTADOS

Foram realizadas 13 oficinas entre julho a setembro de 2023, totalizando 1.232 participantes das áreas de imunização, técnicos dos distritos sanitários de saúde indígena, técnicos de atenção primária e vigilância das capitais, que já levariam o microplanejamento ao seu território. Em algumas oficinas, participaram técnicos de municípios que são polos regionais, a exemplo de São Paulo. Quatro estados tiveram técnicos de todos (ou quase a totalidade) dos seus municípios participando nas oficinas oferecidas pelo Ministério da Saúde: Amazonas, Acre, Amapá e Roraima. 

A oficina de trabalho piloto aconteceu na cidade de Natal, no Rio Grande do Norte, com representantes do estado e de 16 municípios, no contexto da necessidade de se intensificar a vacinação contra febre amarela por conta de epizootias registradas na região, custeada e organizada pela OPAS. Após a experiência no Rio Grande do Norte, consolidou-se o Comitê de Coordenação das Atividades de Vacinação de Alta Qualidade, composto por representantes do PNI, da Secretaria de Atenção Primária à Saúde (APS) e da Secretaria Especial de Saúde Indígena, e houve adaptações da oficina e dos materiais de apoio oferecidos pela OPAS. Como resposta, ofertaram-se aos dois estados (Amazonas e Acre) e aos seus municípios as oficinas de microplanejamento e a antecipação da multivacinação. Antes da publicação dessa portaria, realizaram-se duas oficinas no Amazonas e duas oficinas no Acre, com a presença de técnicos dos estados e de todos os municípios. 

A oficina com maior quantidade de participantes foi a de Manaus (13,3%), seguida de São Paulo (12,7%) e de Curitiba (8,7%) (Tabela 1). A oficina de Goiás, que contou com a presença de pessoas de Goiás, do Tocantins e de Rondônia, foi a que apresentou a menor nota média (4,30) entre os demais estados (Figura 1). Mesmo sendo a menor média, 83,8% assinalaram os 22 itens como bom ou excelente. A oficina com a maior média foi a do Amapá (realizada com o estado do Amapá e seus municípios), com nota de 4,76 e percentual de satisfação de 96,3%. A oficina do Ceará (com a presença de pessoas de Alagoas, Piauí, Ceará e Paraíba) teve média de 4,45, porém 100% de satisfação. Isso indica que todos assinalaram 4 ou 5, ou seja, bom e excelente, mas marcaram mais o valor 4 do que o 5.

**Figura 1:** Nota da avaliação das oficinas realizadas em cada estado, Brasil, 2023

**Tabela 1:** Distribuição por Unidades Federativas (UF) dos participantes nas oficinas realizadas. Brasil, 2023

Número de oficinas	Datas	Local	UF dos participantes	Participantes por UF (n)	Total de participantes, n (%)
1	24 a 28 de abril	Natal	Rio Grande do Norte	68	68 (5,5)
2	8 a 12 de maio	Manaus	Amazonas	158	164 (13,3)
			Roraima e São Paulo	6	
3	18 a 26 de maio	Cruzeiro do Sul e Rio Branco	Acre	89	93 (7,5)
			Rondônia	4	
4	10 a 13 de julho	Macapá	Amapá	63	67 (5,4)
			Maranhão	1	
			Rondônia	3	
5	18 a 20 de julho	Belém	Roraima	18	67(5,4)
			Maranhão	24	
			Pará	25	
6	25 a 27 de julho	Vitória	Rio de Janeiro	31	68 (5,5)
			Espírito Santo	20	
			Distrito Federal	15	
			Rio Grande do Sul	2	
7	31 de julho a 4 de agosto	São Paulo	São Paulo	156	156(12,7)
8	8 a 10 de agosto	Cuiabá	Mato Grosso	44	67 (5,4)
			Mato Grosso do Sul	21	
			Minas Gerais	2	
9	15 a 17 de agosto	Curitiba	Paraná	41	107 (8,7)
			Santa Catarina	25	
			Rio Grande do Sul	36	
			Rio Grande do Norte	3	
			Minas Gerais	2	
10	22 a 24 de agosto	Fortaleza	Alagoas	15	77 (6,3)
			Piauí	20	
			Ceará	20	
			Paraíba	20	
			Rio Grande do Norte	2	
11	29 a 31 de agosto	Goiânia	Distrito Federal	1	101 (8,2)
			Goiás	57	
			Tocantins	28	
			Rondônia	15	
12	4 a 6 de setembro	Salvador	Acre	1	96 (7,8)
			Bahia	54	
			Pernambuco	25	
			Rio Grande do Norte	2	
			Sergipe	14	
13	14 a 14 de setembro	Belo Horizonte	Amazonas	2	101 (8,2)
			Minas Gerais	98	
			Rio Grande do Sul	1	

A menor média entre as dimensões foi para a formação e conteúdo, com nota de 4,38 (Figura 2). Ainda assim, o percentual de satisfação foi alto, com 86,4% dos participantes assinalando como bom ou excelente. Verificou-se diferença pequena para a dimensão que teve maior nota: a de 4,64 para os facilitadores. A satisfação ficou em 94,7%. A avaliação geral dos treinamentos foi 4,57, item que não leva em consideração os demais itens, com satisfação de 95,2%, marcando como bom ou excelente.

**Figura 2:** Avaliação objetiva dos estados onde foram realizadas as oficinas, por dimensão, Brasil, 2023

## DISCUSSÃO

Entre julho e setembro de 2023, foram realizadas 13 oficinas de microplanejamento com 1.232 participantes das áreas de imunização, técnicos de saúde indígena, atenção primária e vigilância de diversas regiões. Estados como Amazonas, Acre, Amapá e Roraima tiveram ampla participação de seus municípios, e oficina realizada em Manaus registrou o maior número de participantes (13,3%). A oficina piloto ocorreu no Rio Grande do Norte, voltada à intensificação da vacinação contra febre amarela. As avaliações gerais foram positivas, com nota média de 4,57 e 95,2% de satisfação, destacando-se os facilitadores com a maior nota (4,64) e formação e conteúdo com a menor (4,38).

No Brasil, os processos de transições demográfica e epidemiológica aparecem como fenômenos interligados e ocorrem justapostos ao desenvolvimento social e econômico do país diferentemente nas macrorregiões, experimentando efeitos diversos sobre a condição de saúde da população. ^12^ Foram realizadas oficinas em distintos estados do Brasil e ofertadas para as 27 Unidades da Federação. O maior número de turma foi em agosto de 2023, totalizando seis, com 578 participantes. Nesse mês, os treinamentos foram realizados em São Paulo, em Mato Grosso do Sul, no Paraná, no Ceará e em Goiás – alguns realizaram a oficina de forma conjunta, pela proximidade geográfica.

A expansão da APS no território brasileiro é notória, com o aumento do número de Unidades Básicas de Saúde, a elevação da cobertura populacional pela Estratégia Saúde da Família e a ampliação de acesso,^14-15^ incluindo na imunização. Ainda coexistem no Brasil desigualdades regionais e questões políticas dificultadoras que influenciam e contribuem para a queda da cobertura vacinal.

O microplanejamento trouxe mudanças efetivas para a sistematização das atividades, como o programa de rotina, as intensificações e a vacinação extramuros (que já era um processo realizado por profissionais da imunização do nível local, mas, sem ser documentado). Esse método tornou possível incorporar a aplicação de critérios e indicadores aos serviços.^8^

As fases do microplanejamento são dinâmicas e, portanto, é possível produzir processos dialéticos para discutir as dificuldades e as facilidades do trabalho em imunização que possam contribuir com a melhoria da cobertura vacinal. Considera-se a realização de plano de ações de microplanejamento por estados e municípios, a ser elaborado com base no reconhecimento da realidade local e da população-alvo, para identificar as ações de vacinação intra e extramuros mais adequadas e eficazes. 

As atividades de vacinação de alta qualidade corroboram e atendem aos critérios desenvolvidos pela OPAS por meio da metodologia de gestão produtiva dos serviços de saúde. Destaca-se que os critérios mencionados abarcam os pilares fundamentais de eficácia, homogeneidade, oportunidade e eficiência. Essa abordagem desempenha um papel central como estratégia para alcançar metas, garantir a avaliação precisa das necessidades e otimização dos recursos disponíveis, além de assegurar o amplo acesso da população à vacinação. ^16^

As oficinas suscitaram discussões e espera-se que a compreensão dos gestores estaduais e municipais levem ao fortalecimento, à incorporação e à priorização das ações em relação à vacinação na rotina dos serviços. Para longo prazo, espera-se a incorporação da estratégia, de forma sustentável, nos estados e municípios brasileiros, a fim de eliminar e controlar doenças preveníveis por vacinação. Verificam-se impacto da sua implantação no incremento na cobertura vacinal e o aumento no número de municípios que atingiram as metas de vacinação, comparando-se os anos de 2022 (pré-microplanejamento) e 2023 (pós-microplanejamento), especialmente em relação à vacina adsorvida difteria, tétano e *pertussis*, hepatite A e tríplice viral. Isso reflete a melhora em comparação com práticas anteriores que apresentavam quedas preocupantes nas coberturas vacinais. ^16^ As oficinas de trabalho foram relevantes nas trocas de experiências entre os diversos atores envolvidos, o que facilitou a integração entre os distintos níveis de gestão, alinhada às necessidades locais. ^16^

A diversidade de profissionais das áreas de vacinação foi capaz de expor questões importantes quanto aos desafios dos estados e municípios para alcance e manutenção de adequada cobertura vacinal. É fundamental, no processo de microplanejamento, que os envolvidos internos e externos estejam engajados para possibilitar momentos reflexivos e de cooperação, capazes de produzir conhecimento e modificar as práticas em saúde, além de aumentar a compreensão de processos críticos que podem estar relacionados com as baixas coberturas vacinais no Brasil. ^17^

Reforçam-se a necessidade de interação e de corresponsabilidade entre as equipes da APS e da vigilância epidemiológica e o apoio entre estados e municípios. Este foi um dos principais pontos observados a partir da análise deste estudo, por ter sido exaustivamente debatido nas oficinas. Esse alinhamento é fundamental para o controle das doenças preveníveis por vacinação.^18^

Como limitação, destaca-se o curto período entre a implementação das oficinas e a avaliação de seus impactos. Ressalta-se que esses achados podem não refletir em mudanças a longo prazo das ações de microplanejamento. Outro ponto foi a dependência da autoavaliação dos participantes, que, embora útil, pode introduzir um viés de resposta positiva.

As oficinas de microplanejamento mostraram-se espaço para sensibilização e troca de experiências entre os atores envolvidos com a vacinação. Essas questões foram capazes de suscitar discussões, e espera-se que levem à incorporação das ações na rotina dos serviços. Isso gera evidência para adoção das atividades de vacinação de alta qualidade e de um regime permanente de vacinação em tais contextos, o que inclui aspectos logísticos e operacionais necessários para implementar e ter sucesso com esse tipo de estratégia. O preenchimento e a discussão dos instrumentos de coleta de dados da oficina possibilitaram aproximar teoria e prática, além de promover a escuta qualificada dos participantes e o relato das necessidades enfrentadas nos serviços de saúde.
